# Total regression of brain metastases in non-small cell lung cancer patients harboring EGFR mutations treated with gefitinib without radiotherapy: two case reports

**DOI:** 10.1186/s13104-015-1834-0

**Published:** 2016-01-02

**Authors:** Masashi Chonan, Norio Narita, Teiji Tominaga

**Affiliations:** Department of Neurosurgery, Iwaki Kyoritsu Hospital, 16 Kusehara, Uchigo Mimaya-machi, Iwaki, Fukushima 973-8555 Japan; Department of Neurosurgery, Kesennuma City Hospital, Kesennuma, Miyagi Japan; Department of Neurosurgery, Tohoku University School of Medicine, Sendai, Miyagi Japan

**Keywords:** Lung cancer, Brain metastasis, Gefitinib, EGFR mutation

## Abstract

**Background:**

Gefitinib is an epidermal growth factor receptor tyrosine kinase inhibitor. Clinical trials have reported its effectiveness in the treatment of brain metastases from non-small cell lung cancer by overcoming the blood–brain barrier. Gefitinib is generally regarded as a relatively safe agent, and several reports have described its efficacy in patients with epidermal growth factor receptor mutation-positive non-small cell lung cancer and a poor performance status.

**Case presentation:**

We herein described two patients with brain metastasis from non-small cell lung cancer who achieved the total regression of metastasis with the administration of gefitinib. A 70-year-old Japanese woman was referred to our hospital with a severe cough. Brain magnetic resonance imaging revealed a metastatic lesion in the left temporal lobe. The tumor was positive for an epidermal growth factor receptor L858R mutation in exon 21 using the peptide nucleic acid-locked nucleic acid polymerase chain reaction clamp method. She was treated with 250 mg gefitinib per day, and, 1 month later, the primary lesion and brain metastasis had totally resolved. A 58-year-old Japanese woman was referred to our hospital with nausea and headache. Brain magnetic resonance imaging revealed a metastatic lesion in the left cerebellar hemisphere and meningeal dissemination. The tumor was positive for the epidermal growth factor receptor L858R mutation in exon 21. She was treated with 250 mg gefitinib per day, and, 3 weeks later, the primary lesion, brain metastasis, and meningeal dissemination had completely resolved.

**Conclusion:**

We successfully treated two lung cancer patients with brain metastasis using gefitinib. Gefitinib therapy may be a suitable treatment for brain metastasis in lung cancer with an epidermal growth factor receptor mutation, particularly in elderly patients with a poor performance status.

## Background

Brain metastasis in lung cancer is a serious clinical condition associated with a poor outcome. Multiple cerebral lesions are associated with a poor prognosis, and the median overall survival from the time of diagnosis is approximately 3–6 months. Whole-brain radiotherapy (WBRT) and platinum-based chemotherapy are the standard therapeutic choices for patients with brain metastases; however, the prognosis of patients with brain metastases is still poor. The optimal treatment for such patients is controversial, and the effectiveness of a combination of radiotherapy, chemotherapy, and epidermal growth factor receptor tyrosine kinase inhibitors (EGFR-TKIs) remains unclear. Systemic chemotherapy is typically ineffective for the treatment of brain metastases because of the blood–brain barrier (BBB). The penetration of chemotherapeutic drugs into the central nervous system (CNS) is primarily limited by the BBB. Gefitinib, an EGFR-TKI, was previously shown to be effective in the treatment of brain metastases from non-small cell lung cancer (NSCLC) by overcoming the BBB. An EGFR mutation is a predictive biomarker for a good response to EGFR-TKIs, even in brain metastases [1]. Gefitinib is generally regarded as a relatively safe agent. Several recent reports described its efficacy in patients with EGFR mutation-positive NSCLC with a poor performance status (PS) [2].

We herein describe two cases of brain metastasis in lung cancer that achieved the total regression of metastasis with the administration of gefitinib.

## Case presentation

### Case one

**Fig. 1:**
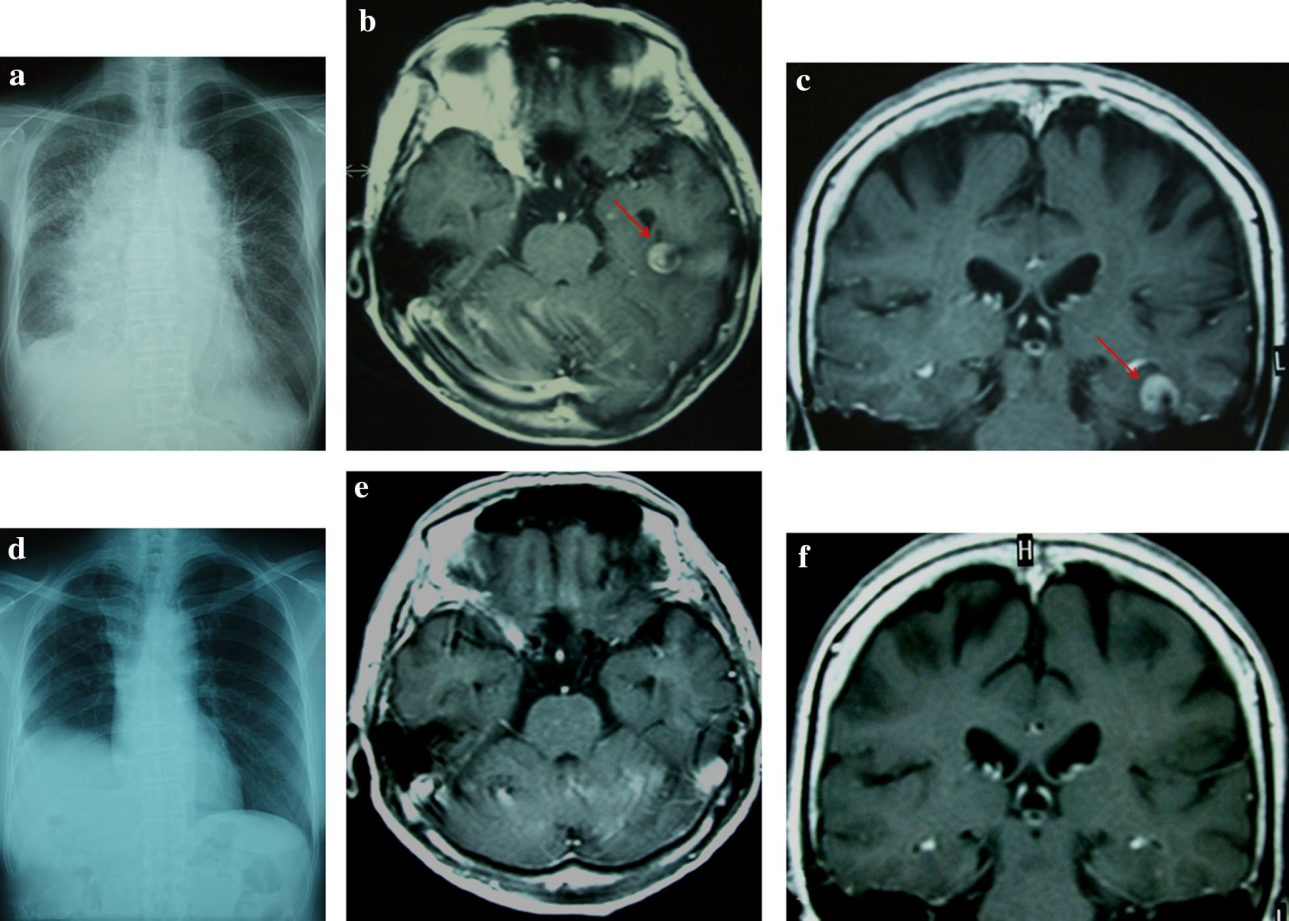
Chest X-ray examination and T1-weighted magnetic resonance imaging (MRI) with gadolinium. **a** *Chest X-ray examination* shows consolidation of the lung and pleural fluid. **b** and **c** Axial and coronal T1-weighted MRI with gadolinium. An enhanced mass lesion (*red arrows*) in the left temporal lobe was observed on admission. **d** The consolidation of the lung and pleural fluid was not detected 1 month after the administration of gefitinib. **e** and **f** Brain metastasis was not detected 1 month after the administration of gefitinib

A 70-year-old Japanese woman without a history of smoking presented at our hospital with a persistent cough, respiratory discomfort, and a chest X-ray abnormality (Fig. 1a). Lung adenocarcinoma was diagnosed based on the results of bronchoscopic biopsy. An EGFR mutation analysis identified the EGFR L858R mutation in exon 21 using the peptide nucleic acid (PNA)-locked nucleic acid (LNA) polymerase chain reaction (PCR) clamp method [3]. Sequential brain magnetic resonance imaging (MRI) revealed an enhancing nodule in the left temporal lobe (Fig. 1b, c). Her PS was poor (PS 3). Gefitinib (250 mg/day) was administered. WBRT was deferred because she did not exhibit any neurological symptoms. Her subjective symptoms improved gradually within 3 weeks of the administration of gefitinib. One month after the initiation of gefitinib, both the primary lesion and metastatic lesion were indiscernible on a follow-up chest X-ray and brain MRI (Fig. 1d, e, f). Thirty-one months after her diagnosis, the patient died due to recurrence of the primary lesion. The recurrence of brain metastasis was not detected. We administrated gefitinib until 1 month before she died. She had undergone best supportive care for 1 month.

### Case two

**Fig. 2:**
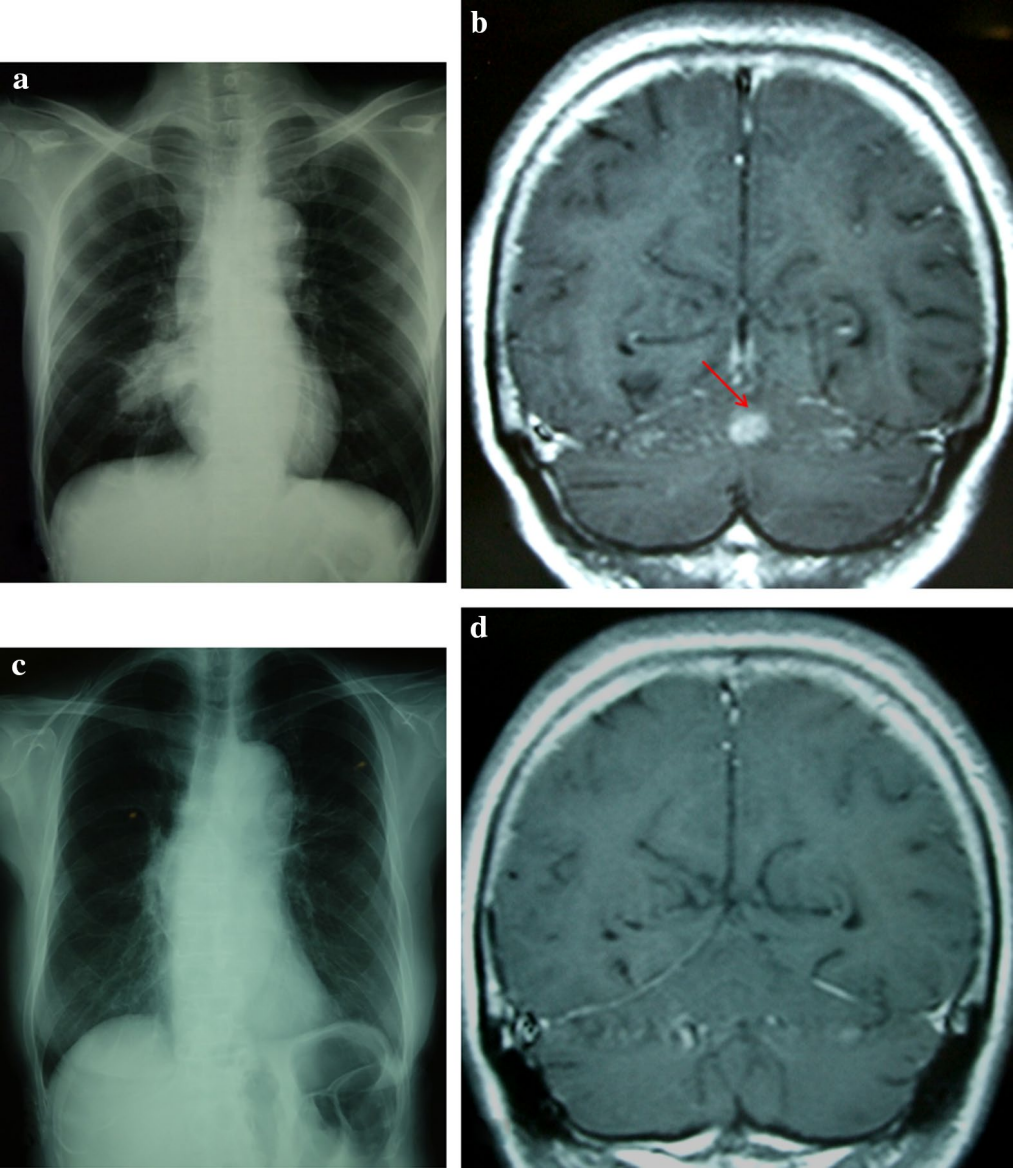
Chest X-ray examination and coronal T1-weighted magnetic resonance imaging with gadolinium. **a** *Chest X-ray examination* shows consolidation of the right lung. **b** Enhanced mass lesion (*red arrow*) and meningeal dissemination were revealed on admission. **c** The consolidation of the right lung was not detected 3 weeks after the administration of gefitinib. **d** Neither brain metastasis nor meningeal dissemination was detected 3 weeks after the administration of gefitinib

A 58-year-old Japanese woman without a history of smoking presented at our hospital with nausea, headache, and a chest X-ray abnormality (Fig. 2a). Lung adenocarcinoma was diagnosed based on the results of bronchoscopic biopsy. An EGFR mutation analysis identified the EGFR L858R mutation in exon 21 using the PNA-LNA PCR clamp method. Sequential brain MRI showed an enhancing nodule in the left cerebellar hemisphere and meningeal dissemination (Fig. 2b). We also examined the cerebrospinal fluid and detected meningeal dissemination. Her PS was poor (PS 4). Gefitinib (250 mg/day) was subsequently administered. WBRT was deferred due to the absence of symptoms. Three weeks after the initiation of gefitinib, both the primary and metastatic lesions were indiscernible on a follow-up chest X-ray and brain MRI (Fig. 2c, d). Nineteen months after the diagnosis, she died due to recurrence of the primary lesion. Neither the recurrence of brain metastasis nor meningeal dissemination was detected. We administrated gefitinib until 2 months before she died. She had undergone best supportive care for 2 months.

## Conclusions

NSCLC with multiple brain metastases is associated with a poor outcome; the overall median survival time of NSCLC was previously reported to be 3.5 months, while the one-year survival rate was 10 % [4]. Therefore, treatment strategies for brain metastasis need to be commensurate with the development of systemic treatments. Standard treatment options include symptomatic therapy with WBRT. The use of conventional chemotherapy for brain metastases has been limited because of a presumed lack of effectiveness due to poor penetration beyond the BBB. The ideal characteristics of compounds with a higher probability of crossing the BBB include a low molecular weight, a non-polar nature, and not being substrates for efflux pumps [5]. Small molecules such as EGFR-TKIs have the ability to cross the BBB. Cappuzzo and coworkers showed that EGFR-TKIs effectively penetrated the BBB because of their chemical structures and low molecular weights [6]. Therefore, these EGFR-TKIs may be efficacious in the treatment of brain metastasis.

Sun and coworkers previously reported that cranial irradiation resulted in lower neurocognitive function in patients without brain metastasis than in those in the observation group [7]. Furthermore, dose per fraction reductions in prophylactic cranial irradiation have been suggested to avoid or reduce the late complications associated with WBRT [8]. Since NSCLC is a relatively radio-resistant malignancy, and WBRT induces late declines in cognitive function, EGFR-TKIs may be a promising option for the treatment of central nervous system metastasis from NSCLC.

Several recent reports described the safety and efficacy of gefitinib in patients with EGFR mutation-positive NSCLC aged >70 years with poor PS [2]. Gefitinib and erlotinib are orally active EGFR-TKIs and show significant efficacy in patients with advanced NSCLC [9]. Since the hematological toxicities of EGFR-TKIs are lower than those of cytotoxic chemotherapy, they may be useful in the treatment of elderly patients and/or patients with poor PS.

In conclusion, NSCLC patients with brain metastases generally have poor PS. Due to its ability to reverse poor PS and achieve the total regression of brain metastases, EGFR-TKI therapy may be a suitable treatment for brain metastasis in lung cancer with an EGFR mutation, particularly in elderly patients with poor PS.

## Consent

Written informed consent was obtained from the patients for publication of this Case Report and any accompanying images.

## Abbreviations

WBRT: whole-brain radiotherapy
EGFR-TKIs: epidermal growth factor receptor tyrosine kinase inhibitors
BBB: blood–brain barrier
CNS: central nervous system
NSCLC: non-small cell lung cancer
PS: performance status
PNA: peptide nucleic acid
LNA: locked nucleic acid
PCR: polymerase chain reaction
MRI: magnetic resonance imaging
